# Strategies for oral delivery of bioactive peptides with focus on debittering and masking

**DOI:** 10.1038/s41538-023-00198-y

**Published:** 2023-05-26

**Authors:** Armin Mirzapour-Kouhdasht, David Julian McClements, Mohammad Sadegh Taghizadeh, Ali Niazi, Marco Garcia-Vaquero

**Affiliations:** 1 School of Agriculture and Food Science, University College Dublin, Dublin, 4 Ireland; 2 Department of Food Science, University of Massachusetts, Amherst, MA 01003 USA; 3 Institute of Biotechnology, Shiraz University, Shiraz, Iran

**Keywords:** Peptides, Agriculture

## Abstract

Protein hydrolysis is a process used in the food industry to generate bioactive peptides of low molecular weight and with additional health benefits, such as antihypertensive, antidiabetic, and antioxidant properties that are often associated with their content on hydrophobic amino acids. This results in an increased bitterness of the products, making them less desirable for their use in food formulations. This review summarizes the main dietary sources of bitter bioactive peptides, including methods to determine their bitterness, such as the Q-values and electronic tongue; and the main factors and mechanisms underlying the bitterness of these compounds. The main strategies currently used to improve the taste and oral delivery of bioactive peptides are also discussed together with the main advantages and drawbacks of each technique. Debittering and masking techniques are reported in detail, including active carbon treatments, alcohol extraction, isoelectric precipitation, chromatographic methods, and additional hydrolytic processes. Other masking or blocking techniques, including the use of inhibitors, such as modified starch, taurine, glycine, and polyphosphates, as well as chemical modifications, such as amination, deamination, acetylation, or cross-linking were also discussed. The findings of this work highlight encapsulation as a highly effective method for masking the bitter taste and promoting the bioactivity of peptides compared to other traditional debittering and masking processes. In conclusion, the article suggests that advanced encapsulation technologies can serve as an effective means to mitigate the bitterness associated with bioactive peptides, while simultaneously preserving their biological activity, increasing their viability in the development of functional foods and pharmaceuticals.

## Introduction

**Fig. 1:**
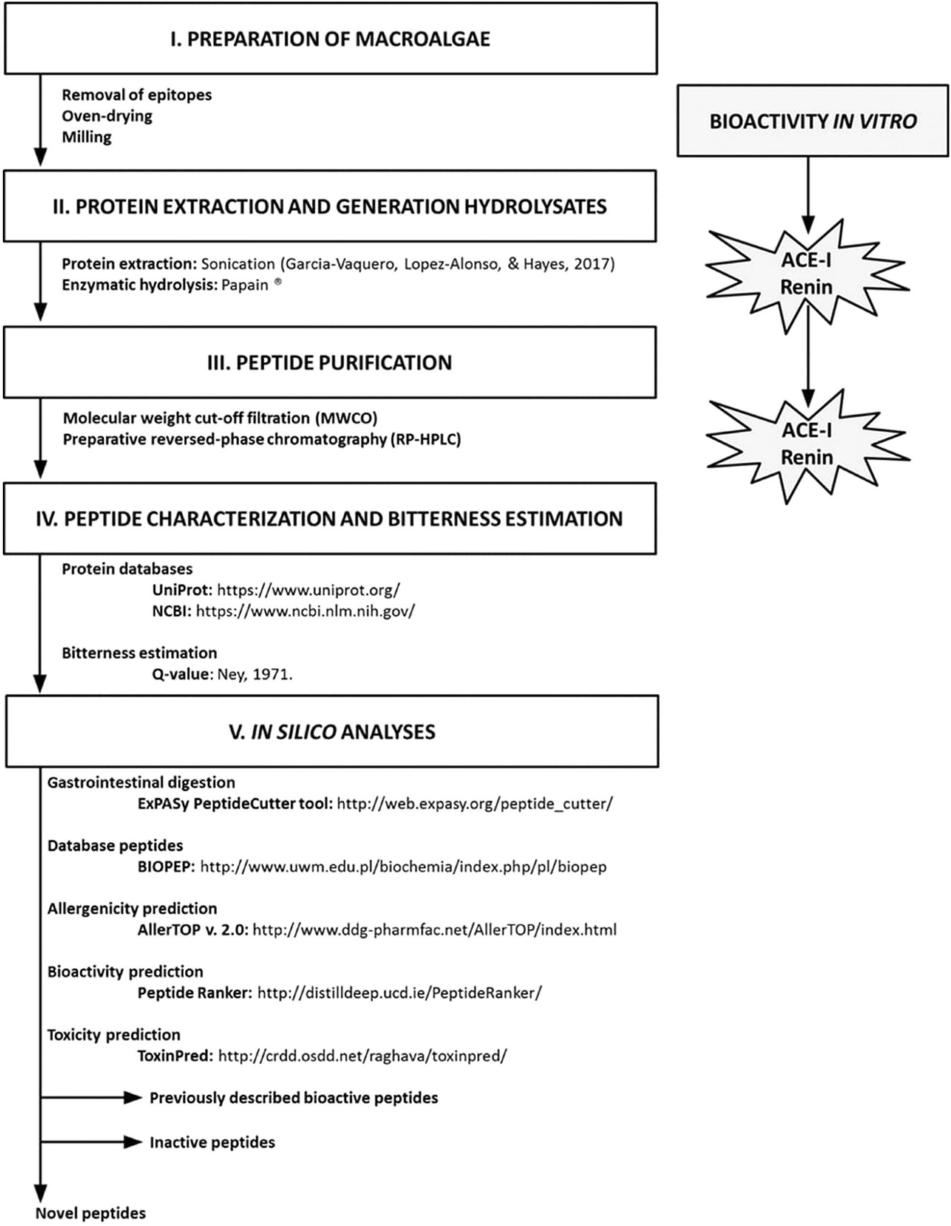
Scheme of a proposed strategy for the generation of bioactive peptides from macroalgae^21^.

Research has shown that bioactive peptides exhibit a range of potentially beneficial biological activities, which has stimulated interest in their application as therapeutic agents. In the United States, more than 60 peptides exhibiting therapeutic properties have been approved for human consumption since 2018. One of the most significant peptide-based medications licensed and commercialized since the 1920s is insulin^1^. The World Health Organization (WHO) has stated that “non-communicable illnesses including cancer, diabetes, and hypertension cause 36 million fatalities annually”^2^. Studies have shown that bioactive peptides may be able to reduce the risk of a range of these chronic illnesses, including diabetes^3–6^, hypertension^7,8^, and cancer^9–13^. Consequently, the development of functional foods, supplements, or drugs containing bioactive peptides may be able to improve the health of the general population and strategies for the generation of these compounds from different protein sources, such as macroalgae, have been proposed (Fig. 1). However, any bioactive formulation intended for oral administration should be appealing to consumers^14^, which means it should not have an undesirable flavor profile or mouthfeel.

The bitter taste of many bioactive peptides is one of the largest hurdles to their widespread use in functional foods, supplements, and drugs intended for oral ingestion^15,16^. Many animals, including humans, perceive peptides as having an unpleasant flavor due to millions of years of evolution, since peptides are often associated with harmful substances^17^.

This article reviews the different food sources of bioactive peptides and the various strategies that have been developed to make them more palatable, including debittering, masking, modulation, and encapsulation technologies. In addition, it considers the application of these technologies in the development of functional foods, supplements, and pharmaceuticals intended for oral administration.

## Generation of bitter peptides from different protein sources

According to Ney^18^, the bitterness of peptides is closely linked to their hydrophobicity. The sum of the free energy arising from the transfer of each amino acids’ side chains from ethanol to water divided by the total number of amino acid residues in the peptide is known as the average hydrophobicity of a peptide or Q-value:

1 $$Q=\sum \frac{\triangle G}{n\,}$$

Here, Δ*G* and *n* are the transfer free energy and number of amino acid residues, respectively. When the Q value of a peptide reaches 1400 cal/mol, it is regarded as being bitter^19^, whereas a Q value of less than 1300 cal/mol denotes a non-bitter peptide. An intermediate value suggests that the peptide has a slight bitterness. In addition, the Q-value may be used to assess the bitterness of peptides with molecular weights of less than 6 kDa, roughly 55 amino acid residues^20^. In a study conducted by Garcia-Vaquero et al.^21^, bioactive peptides from *Ulva lactuca* were produced using in vitro and in silico methods. Except for the sequences SAGVLPWK, GAAPTPPSPPPATKPSTPPKPPT, IECCLLFALV, PVGCLPK, DAVEIWRVK, DEVIPGAL, PKPPALCN, and PPNPPNPPN, all of the detected peptides had Q-values below the bitterness threshold. The Q-values of these peptides ranged from 1440 to 1743 cal/mol, which suggests that they had a bitter taste. According to Fan et al.^22^, Alaska pollock frame hydrolysates showed no bitterness when the Q-value was below 5.44 kJ/mol, but did exhibit bitterness when the Q-value was higher than 5.86 kJ/mol. In addition, hydrolysates made from high Q-value proteins like casein (1605 cal/mol), soy protein (1540 cal/mol), and zein (1480 cal/mol) also have a bitter taste^23^.

The ratio of hydrophilic to hydrophobic amino acids determines how soluble a protein is. In this regard, hydrophobic peptides, which have a bitter taste, are released more by water-insoluble proteins. It has been demonstrated that peptide bitterness is strongly related to the amount of hydrophobic amino acids present, and thus the peptide’s overall hydrophobicity^24^. Therefore, the release of bitter peptides from protein is related to the amino acid composition and sequence, enzyme active site, as well as degree of hydrolysis^25^. When hydrolyzing a water-insoluble protein under identical conditions, alcalase and trypsin produce different types of peptides. Trypsin cleaves peptide bonds at the C-terminal with basic residues like arginine and lysine, while alcalase is an endopeptidase that positions hydrophobic residues such as phenylalanine, tyrosine, and tryptophan in the middle of the peptide chain, resulting in hydrophobic residues at the end of the peptide chain. This difference in enzyme specificity leads to the production of bitter and non-bitter peptides. To put it differently, hydrolysis by alcalase leads to the production of peptides with a bitter taste as a result of the hydrophobic residues being present at the end of the protein hydrolysates^26,27^.

It is possible to adjust the conditions used to hydrolyze proteins to produce bioactive peptides that are not bitter, such as hydrolysis method, time, temperature, or solution conditions. For instance, Fan et al.^22^ reported that the Q-value of hydrolysates increased during the first 90 min of enzymatic hydrolysis but then decreased at longer times. In the earlier stages of protein hydrolysis, the hydrophobic amino acids generated are relatively large and can interact with the bitterness receptors on the tongue^28–30^. In contrast, during the later stages of protein hydrolysis, only small peptides or free amino acids are present, which do not strongly interact with the tongue^28,31^. Table 1 summarizes the taste of each amino acid to establish clear comparisons.

**Table 1:** The taste qualities of amino acids and sensing mechanisms by G protein-coupled receptors (GPCRs).

Amino acid	Human taste perception	Sensing mechanism by GPCRs
ʟ-Alanine	Sweet	GPRC6A
ʟ-Arginine	Bitter	GPRC6A
ʟ-Asparagine	Bitter	T1R1/T1R3
ʟ-Aspartic acid	Umami, sour	T1R1/T1R3
ʟ-Cysteine	Sulfurous	T1R1/T1R3
ʟ-Glutamic acid	Umami, salty	T1R1/T1R3, mGluRs
ʟ-Glycine	Sweet	GPRC6A
ʟ-Histidine	Bitter	CaSR
ʟ-Hydroxyproline	Sweet	T1R1/T1R3
ʟ-Isoleucine	Bitter	T1R1/T1R3
ʟ-Leucine	Bitter	T1R1/T1R3
ʟ-Lysine	Bitter, salty, sweet	GPRC6A
ʟ-Methionine	Bitter, sulfurous	GPRC6A
ʟ-Phenylalanine	Bitter	CaSR
ʟ-Proline	Sweet	T1R1/T1R3
ʟ-Serine	Sweet	GPRC6A
ʟ-Threonine	Sweet	T1R1/T1R3
ʟ-Tryptophane	Bitter	CaSR
ʟ-Tyrosine	Bitter	Not detectable yet
ʟ-Valine	Bitter	T1R1/T1R3

The information summarized in this table was compiled from San Gabriel and Uneyama^198^ and Wellendorph and Bräuner‐Osborne^199^.

GPRC6A: G-protein-coupled receptor family C, group 6, subtype A, T1R1 and T1R3: Taste 1 Receptors, mGluRs: metabotropic glutamate receptors, CaR: calcium-sensing receptor.

To a first approximation, the bitterness of many bioactive peptides can be predicted from their Q-value, which mainly depends on their hydrophobicity. However, researchers have shown that the bitterness of peptides also depends on their molecular weight, amino acid sequence, and composition^28,32,33^.

Cultural and regional differences affect how people perceive bitterness, making it challenging to develop standardized methods to measure bitterness accurately^34^. The electronic tongue (E-tongue) can be used to overcome this problem and efficiently screen peptides from different sources and hydrolysis methods in a standardized manner. For instance, Nath et al.^35^ used an E-tongue to evaluate the bitterness of bioactive peptides derived from tryptic and ferment milk protein concentrate (MPC). The MPC was hydrolyzed using the tryptic hydrolysis method followed by hydrolysis using two microbial species utilized in yogurt manufacturing (*Lactobacillus bulgaricus* and *Streptococcus thermophilus*). The results of the experiment indicated that there is a direct relationship between the bitterness of the hydrolysates and the concentration of trypsin; and the potential application of the E-tongue was demonstrated by measuring the bitterness of peptides at each hydrolysis step using a standard model solution of quinine. These methods are particularly useful for rapidly screening peptides produced from different sources and different hydrolysis methods. Ideally, researchers would like to use these methods to identify peptides that have a high bioactivity but a low off-flavor. In the remainder of this section, we highlight different sources of food proteins that can be used to produce bioactive peptides.

### Milk proteins

The hydrolysates from milk proteins have been shown to exhibit a range of biological activities, which makes them suitable for application in functional food products^36^. Milk protein hydrolysates are commonly derived from their parent proteins using either enzymatic digestion or fermentation processes^37^. Hydrolysates have valuable biological activities but are sometimes limited in use due to their bitter taste^38^, which is influenced by various factors such as the nature of side chains on the peptides produced by hydrolysis, distribution and location of bitter taste residues, hydrophobicity, degree of hydrolysis, amino acid conformation, peptide sequence, and carbon number on the amino acid side chain^16,33,39–42^. Whey protein hydrolysates have malty, brothy potato, animal, and bitter flavors^43^, while sodium caseinate generates more bitter peptides during hydrolysis than whey protein^44,45^.

Researchers have reported that increasing the degree of hydrolysis of proteins by more than 8% leads to the production of bitter-tasting peptides^46^. Nevertheless, hydrolysis can also reduce the allergenicity of milk proteins, such as casein, β-lactoglobulin, and ɑ-lactalbumin, increase the production of bioactive peptides, and improve the nutritional quality of these products. Consequently, the potential advantages of hydrolysis of dairy proteins should not be ignored.

The type of enzyme used to carry out the hydrolysis of proteins affects the types and amounts of bitter peptides produced. For instance, hydrolyzing whey proteins using alcalase 2.4 L was reported to generate more bitter peptides than hydrolyzing them using prolyve or corolase under the same reaction conditions^47^. The amino acid sequence of peptides has also been shown to strongly influence their bitterness. For instance, Shinoda et al.^42^ reported that inverting the peptide sequence of RGPFFIIV (derived from beta-casein) greatly decreased its bitter taste. Interestingly, it has been reported that some animals avoid consuming casein hydrolysates due to the presence of bitter peptides^16^. Therefore, methods are needed to reduce the bitterness of these hydrolysates if they are going to be utilized as bioactive ingredients in food and feed applications.

### Soybean proteins

Soybeans contain relatively high concentrations of good quality proteins. Unfortunately, eight proteins in soybean (Gly m1-Gly m8) have so far been reported to be allergens, which limits their use as a protein source^48^. The conversion of soybean proteins into hydrolysates and peptides can overcome this limitation. In addition to being used as bioactive ingredients in functional foods, soybean protein hydrolysates are also widely used as techno-functional ingredients, such as thickeners, gelling agents, emulsifiers, and foaming agents^49,50^. Again, however, one of the most important factors limiting their use in many food and beverage applications is their bitter taste (Table 2). Cho et al.^32^ reported that the bitterness of soybean protein hydrolysates is associated with their molecular mass, with larger peptides (>4 kDa) being more bitter than smaller ones (<1 kDa). However, it has been reported that the cause of bitterness in alcalase-treated soybean hydrolysates was due to the presence of 1 kDa hydrophobic peptides^51^. The bitterness of protein hydrolysates has been reported to depend on the type of enzyme used to hydrolyze them, with the bitterness decreasing in the following order: alcalase > neutrase ≈ trypsin > Flavourzyme^52^. However, in another study, the bitterness of bromelain-treated soybean hydrolysates (4% hydrolysis) was reported to be no different from that of soybean protein isolate^53^. In contrast, another study showed that soybean hydrolysates generated using bromelain (10–15% hydrolysis) were extremely bitter^52^. Furthermore, it has also been suggested that hydrophobic amino acids, such as leucine and phenylalanine, do not contribute to the bitter taste of soy hydrolysates^54^. Dall Aaslyng et al.^55^ indicated that soybean hydrolysates exhibit a bitter taste when heated, which appeared to be due to pyrazines. Furthermore, bitter peptides were reported from miso (salted and fermented soybean paste), natto (fermented soybean), and soy sauce^56–59^. Consequently, many factors appear to contribute to the bitterness of soy protein hydrolysates.

**Table 2:** Q-values of peptides produced during the hydrolysis of various proteins using different hydrolysis conditions.

Protein source	Identified peptide sequences (Q value)^a^	Hydrolysis conditions^b^	Bitterness evaluation method	Factors influencing bitterness	Bioactivities	References
Milk	VLPVPQK (1876), EIVESLSSSEESITRINK (972), FLLY (2189), PFPIIV (2810), SDISLLDA (1171), TTMPL (1324), ESISSSEEIV (983), FPQY (2088), PPFL (2707), IESPPEIN (1660), DERFFSDK (1233), MMSFV (1591), PTPEG (1272), TTMPLW (1739), MPFPK (2383), VLSR (1185), SDISLLDA (1171), LLFCME (1814), NLPPLTA (1551), SFLY (1918), LCVLH (1683), KHQGL (890), KEGI (1341), KKNQDKTEI (1001), DAQSAP (825)	Bromelain (50 °C, pH = 7), proteinase K (37 °C, pH = 7), papain (65 °C, pH = 7), and ficin (50 °C, pH = 6.5); E/S: 1/100 (w/w); time: 3 h	BIOPEP-UWM database prediction	Hydrophobicity and enzyme active site	Antioxidative, opioid, immunomodulating, ACE inhibitory	^200^
	LLYQEPVLGPVRGPFPIIV (1933), LYQEPVLGPVRGPFPI (1848), LYQEPVLGPVRGPFP (1760), LYQEPVLGPVRGPFPIIV (1921), LLYQEPVLGPVRGPFP (1784), FFVAPFPEVFGK (2000)	Flavourzyme (50–55 °C, pH = 5.5–7.5), protamex (50 °C, pH = 7–8), promod 523MDP^TM^ (45–55 °C, pH = 5–7), and flavorpro 937MDP^TM^ (50 °C, pH = 5–7); time: 9 h	Taste panelists	Degree of hydrolysis	–	^201^
	–	Trypsin (40 °C); E/S: 0.008–0.032 g/L; time: 10 min	Taste panelists	Hydrophobicity	–	^35^
	VEELKPTPEGDLEIL (1452), VEE (1009), LKPTPE (1685), GDLE (831), IL (2660)	Alcalase 2.4 L, prolyve 1000 and corolase 7089; 50 °C; pH = 7, E/S: 0.25%	Taste panelists	Hydrophobicity	–	^47^
	VLVLDTDYK (1487), GLDIQK (1234), IDALNEK (1269), TPEVDDEALEK (1109), VGINYWLAHK (1684), HIRLS (1374), KTKIPAVF (1845), MAASDISLL (1247), VRTPEVDDE (1075), LVRTPEVDDE (1183), VRTPEVDDEALE (1102), LVRTPEVDDEALE (1182), VLPVPQ (1922), KAVPYPQ (1802), RDMPIQAF (1580), SQSKVLPVPQ (1297), PEGDLEI (1426), VEELKPT (1378), VEELKPTPE (1460), AVPYPQ (1835)	Alcalase (65 °C, pH = 8.5, E/S: 1/100, time: 5 h), corolase PP (45 °C, pH = 7.5, E/S: 1/50, time: 7 h), flavourzyme (50 °C, pH = 6.5, E/s: 1/100, time: 8 h)	Taste panelists	Enzyme active site	Antioxidative	^202^
	–	Alcalase (55 °C, pH = 8.5), protamex (50 °C, pH = 6.5), flavourzyme (50 °C, pH = 7); E/S: 1/50; time: 30–180 min	E-tongue measurement	Enzyme active site	–	^26^
	–	Commercial protein hydrolysates	Taste panelists	Degree of hydrolysis and hydrophobicity	–	^43,203^
Soybean	FLS (1666), LLPH (2000), INGY (1458), IYIG (2253), VYDV (1734), SVIY (1913), VYFV (2309), ISIY (2245), VVLY (2126), DIF (2211), GYPVV (1851), YVVL (2126), SGFTL (993), SNLNFL (1188)	Alcalase (50 °C, pH = 8, E/S: 12 AU/Kg)	Taste panelists	Hydrophobicity	–	^204^
	LSVISPK (1657), DVLVIPLG (1829), LIVILNG (1781), NPFLFG (1800), ISSTIV (1348), PQMIIV (2102), PFPSIL (2328), DDFFL (1815), FFEITPEK (1823)	Bromelain (50 °C, pH = 7), proteinase K (37 °C, pH = 7), papain (65 °C, pH = 7), and ficin (50 °C, pH =6.5); E/S: 1/100 (w/w); time: 3 h	BIOPEP-UWM database prediction	Hydrophobicity and enzyme active site	Antioxidative, opioid, immunomodulating, ACE inhibitory	^205^
	AFPGSAKDIENLIK (1443), YVVNPDNNENLRLITL (1297), RRPSYTNGPQEIY (1253), SLENQLDQMPRRF (1092), VVNPDNNENLRLITL (1205), RIDPELEKEIGAK (1425), ENQLDQMPRRF (1099), SLLNALPEEVIQHTFNLK (1311), NQLDQMPRRF (1149), RAELSEQDIFVIPA (1437), LVPPQESQRR (1171), IIDTNSLENQLDQMPRRFYLA (1271), GINAENNQRNFLA (848), KLHENIARPS (1275), IVRNLQGENEEEDSGAIVTVK (950), LSIVDMNEGALLLPHFNSK (1332), AGANSLLNALPEEVIQH (1094), RVSDDEFNNY (988), NALEPDHRVESE (966), LAGNPDIEHPETM (1237), GKHQQEEENEGGSIL (656), EQGGEQGLEYVVF (997)	Protex 6 L (55 °C, pH = 8), protease A 2 SD (50 °C, pH = 7); E/S: 0.5%; time: 1–5 h	Taste panelists	Hydrophobicity and enzyme active site	Antioxidative	^24^
Corn	–	Flavourzyme (50 °C, pH = 7), alcalase (50 °C, pH = 8), neutrase (50 °C, pH = 7), papain (55 °C, Ph = 7), and trypsin (40 °C, pH = 7.5); E/S: 3%; time: 10–100 min	Taste panelists	Degree of hydrolysis and hydrophobicity	ACE inhibitory	^65^
Fish	KAEPAPAPAPAPAPAPAPAP (1775), DEKSPAMPVPGPM (1558), GPAGPRGPSGERGEVGPA (986), LDKNKDPLNDSVVQ (1120), FAGDDAPRA (1125), SGPPVPGPIGPM (1759), LGAGDTDGDGKIGAD (721), DEKSPAMPVPGPMGPM (1549), IEDPFDQDDWEH (1415), LEQQVDDLEGSLE (846), APDPGPGPMG (1461), NWDDMEKIW (1754), GPPVPGPIGPM (1922), ERLEDEEEIN (963), AVIDQDKSGFIEEDELKLF (1349), RDLTDYLMK (1346), MPVPGPMGPM (1834), GPPVPGPIGPMG (1762), LGEQIDNLQRVK (1054), DPFDQDDWEHWAK (1495), GVDNPGHPF (1321), EDDIHPRNPPKFD (1558), MDEPVVVPGKPY (1775), GWLDKNKDPLNDSVVQ (1218), FIEEDELKLF (1719), SVDDEFPDLTK (1241), AGDDAPRAVFPS (1236), DRPGPPDGPLE (1480), SPAMPVPGPM (1747), GPSGKDGPRGPRGD (928), GEEGKRGPTGEIG (784), LDLPGPPIGPIN (1901), DLPGPPIGPIN (1878), GPAGPRGPSGA (1004), PPVPGPIGPM (2114), DRPGPPDGPLEVK (1517)	Neutrase (50 °C, pH = 7, E/S: 0.4–0.8%), bromelain (50 °C, pH = 7, E/S: 0.6–0.8%), flavourzyme (50 °C, pH = 7, E/S: 0.8%), protamex (50 °C, pH =7, E/S: 0.4–0.6%), papain (55 °C, pH = 7, E/S: 0.6–0.8%), and alcalase (55 °C, pH = 9, E/S: 0.6–0.8%); time: 2–10 h	Taste panelists	Hydrophobicity and enzyme active site	–	^25^
	–	FoodPro PNL enzyme, 55 °C, E/S: 0.17–0.22 w/w, time: 50 min	Taste panelists	Hydrophobicity and enzyme active site	–	^206^
	–	Papain (pH = 7), trypsin (pH = 8), pepsin (pH = 2), alkaline (pH = 10) and flavourzyme (pH = 6); 50 °C; E/S: 100 U/g; time: 150 min	Taste panelists	Enzyme active site	Leucocytes and lysozyme activity	^207^
	–	Protamex (40 °C, pH = 6), alcalase (60 °C, pH = 8), trypsin (38 °C, pH = 8), flavourzyme (50 °C, pH = 6) and neutrase (45 °C, pH = 7); E/S: 2%; time: 5 h	Taste panelists	Hydrophobicity and enzyme active site	–	^208^
Seaweed	–	Protease A ‘Amano’ G (50 °C, pH = 7), protease M ‘Amano’ (50 °C, pH = 4.5), protease N ‘Amano’ (55 °C, pH = 7), protease P ‘Amano’ 3G (45 °C, pH = 8), protease S ‘Amano’ (70 °C, pH = 8), blomelain F (60 °C, pH = 9), proleather FG-F (60 °C, pH = 10), peptidase R (45 °C, pH = 7), umamizyme (50 °C, pH = 7), newlase F (45 °C, pH = 3), papain W-40 (65 °C, pH = 7), pancreatine F (45 °C, pH = 9), pepsin (45 °C, pH = 2), denazyme AP (50 °C, pH = 7), denapsin 10P (50 °C, pH = 3), XP-415 (55 °C, pH = 3), alcalase 2.4 L FG (60 °C, pH = 7), E/S: 1%; time: 18 h	–	Hydrophobicity	ACE inhibitory	^209^
Synthetic peptides	YGLF (1926), YPFPGPIPN (2257), IPAVF (2318), LLF (2393)	–	Taste panelists	Hydrophobicity	–	^121^
	MPFPKYPVEPF (2336), GPVRGPFPIIV (2034), MAPKHKEMPFPKYPVEPF (1979), APHGKEMPFPKYPVEPF (1905)	–	Taste panelists	Hydrophobicity	–	^210^
Bovine muscle	–	Alcalase (50 °C, pH = 7.5), flavourzyme (50 °C, pH = 7), protamex (55 °C, pH = 7.5), neutrase (50 °C, pH = 7), proteAX (50 °C, pH = 7), protease P 6 SD (40 °C, pH = 7), protease A 2 SD (50 °C, pH = 7), papain P1 (55 °C, pH = 7), bromelain (40 °C, pH = 7) and sumizyme BNP-L (50 °C, pH = 7); E/S: 0.5%; time: 1–5 h	Taste panelists	Degree of hydrolysis and enzyme active site	–	^150^
Porcine plasma	–		Taste panelists			
Bovine hemoglobin	VVYPWTQR (1715), VVYPW (2614)	Pepsin (37 °C, pH = 3)	Taste panelists	Hydrophobicity	–	^211^
Pea	–	Alcalase (50 °C, pH = 8), flavourzyme (50 °C, pH = 7), papain (40 °C, pH = 6.5), trypsin and α-chymotrypsin (37 °C, pH = 8); E/S: 4%; time: 4 h	Taste panelists	Hydrophobicity and enzyme active site	Antioxidative, ACE inhibitory	^84^
	–	Alcalase (55 °C, pH = 8.5, E/S: 3%, time: 2.5 h) followed by protemax (50 °C, pH = 7, E/S: 1%, time: 1 h)	Taste panelists	Hydrophobicity	ACE and DPP-IV inhibitory	^87^
Peanut	–	Multifect PR 6 L and flavorzyme (pH = 9.5), protamex, neutrase, and Papain (pH = 7.5); 45 °C	E-tongue measurement	Degree of hydrolysis	–	^212^
Soy, pea, and canola	–	Flavourzyme 1000 L, protease P “Amano” 6 SD, deltazymAPS-M-FG, promod278, proteAX-K, and peptidase R; pH = 7.3; 50 °C; E/S: 2%; time: 2 h	Taste panelists	Hydrophobicity and enzyme active site	–	^213^
Chickpea	LR (1465), PLLVE (1926), SPKAGAGK (959), HATGGGSGR (257), PHPATSGGGL (949), TPKASATAAL (976), TLTTGTGGLL (632), YVDGSGTPLT (1005), TKTPGAGTSAGL (677), KEGGGTGTGAAR (376), STGPNAGGGAGGY (546), TLLFTELLF (1654), KNGAAGPSTVAR (787), LASEGASAATGAF (733), VLTSGAGSGAAALT (658), KNGLGAGAGAGSAR (549), LSAHAGGTGATLW (863), LDLARAGGCPTKN (1015), SPQSPPFATPLW (1754), LLSASMGSQLLSF (1053), GKGSGAF (750), TRGTGGR (214), KMTAGSGVT (642), KSGGGGGGTAVT (345), GKAAPGSGGGTKA (649), RASAAGGGGGGVSSR (380), GKGSSGTGAGGASVSGVT (372), NKKSGAGGGSGAGKGGVA (498), LLGELCGSGNTVVEL (991), QNPLSSAAPTGAGKPY (1082), GLTQGASLAGSGAPSPLF (961), AMMELGWSTSGEFLL (1220)	Pepsin/pancreatin (E/S: 1/50), bromelain (55 °C, pH = 5.5–6.5, E/S: 1/50, time: 30 min)	BIOPEP database prediction	Hydrophobicity	DPP-IV, DPP-III, ACE, renin, α-glucosidae and α-amylase inhibitory, Antiamnestic, antithrombotic, antioxidative, hypolipidemic, HMG-CoA reductase	^214^
Flaxseed	–	Alcalase (3000 U/g, 60 °C, pH = 8), flavourzyme (120 U/g, 50 °C, pH = 6.5); time: 2 h	Taste panelists	Degree of hydrolysis, hydrophobicity, and enzyme active site	–	^215^
Cricket and mealworm	–	Flavourzyme 1000 L, protease P “Amano” 6 SD; pH = 7.3; 50 °C; E/S: 2%; time: 2 h	Taste panelists	Enzyme active site	–	^216^

^a^Values in brackets are Q values calculated following the formula as described in Eq. 1.

^b^Abbreviation E/S indicates the ratio of enzyme to substrate.

### Corn proteins (Zein)

Zein is one of the most important proteins in corn, accounting for around 65% of the total corn gluten^60^. One of the biggest limitations to the use of this protein in the food industry is its poor solubility in water due to the presence of high amounts of non-polar amino acids, such as alanine, leucine, and proline^61^. For this reason, enzymatic hydrolysis has been investigated as a means of increasing the solubility and functionality of zein^62–64^. Hydrolysis of corn gluten results in the production of bioactive peptides (Table 2) containing hydrophobic residues, such as leucine, isoleucine, valine, tyrosine, phenylalanine, and tryptophan, which make them bitter^65^. The addition of salts due to the adjustment of pH during the hydrolysis of corn proteins exposes amino acid hydrophobic groups, contributing to the generation of bitter bioactive peptides. In contrast, the production of corn hydrolysates and peptides using microbial fermentation decreases the production of bitter peptides^64^. Consequently, the hydrolysis conditions for zein should be optimized to generate bioactive peptides that have a low bitterness.

### Fish proteins

Fish is another major source of proteins in the human diet^66^. The fishing industry generates many by-products that are typically converted into low-value commodity products, such as fish meal, fertilizers, and animal feed^67^. These protein-rich by-products typically make up about 60–70% of the weight of live fish^68^. Hydrolyzing these by-products can be used to produce bioactive peptides, thereby converting a low-value product into a high-value one^68^. Similar to the other proteins described above, the hydrolysis of fish proteins is accompanied by the production of bitter peptides (Table 2). The hydrophobicity of the peptides produced from fish has been shown to be a key factor influencing their bitterness^69^ and it is generally described well by the Q-rule introduced by Ney^23^ discussed earlier. Degree of hydrolysis is a critical factor for bitterness of fish protein hydrolysates. Increasing it generates more hydrophobic peptides and increases bitterness, as observed in salmon skeletal protein hydrolysis by alcalase^70^.

Another factor that affects the bitterness of fish hydrolysates is the type of enzyme used. For instance, the hydrolysis of Alaska Pollock frame using 10 different enzymes resulted in the production of hydrolysates with molar masses below 1355 Da^71^. The hydrolysates with the lowest bitterness were generated using MEAP, a mixture of animal proteases with both endo- and exopeptidase activities^71^. Lalasidis et al.^72^ indicated that the hydrolysis of fish proteins using alcalase followed by pancreatine did not produce bitter hydrolysates. Amino acid sequences can also affect the bitterness of fish hydrolysates. For instance, reversing the sequence of a peptide has been shown to reduce its bitterness^42^. Furthermore, the molecular weight distribution of fish protein hydrolysates can affect their bitter taste. Atlantic salmon proteins from by-products were hydrolyzed using five enzymes, which resulted in hydrolysates with acceptable bitterness at degrees of hydrolysis above 25%, which contained peptides below 2 kDa^73^. Mackie^74^ reported no bitter taste in fish hydrolysates containing peptides larger than 6 kDa, even for peptides with a Q-value exceeding 1300. However, there are several reports on the presence of bitter peptides in fish sauce and dried fish flakes (katsuobushi)^75,76^.

### Seaweed proteins

Seaweed consumption has been increasing over the past two decades, with a market growth of 20% during this period. Seaweeds are a good source of high quality protein compared to traditional terrestrial crops^77^. According to a study by Fleurence^78^, the protein content of red seaweeds (35 to 47% of dry weight) is higher than those of green seaweeds (10–26% of dry weight) followed by brown seaweeds (3–15% of dry weight). Therefore, red seaweeds may be particularly useful for the generation of peptide ingredients. The protein fraction used for this purpose is often a by-product of traditional seaweed processing operations, such as the isolation of functional polysaccharide ingredients (like carrageenan or alginate).

Seaweed protein hydrolysates exhibit a wide range of functional activities^79^. Hydrolyzed seaweed has been reported to have a desirable umami taste, as well as an undesirable bitter taste (Table 2). Laohakunjit et al.^80^ reported that the bitter taste of seaweed hydrolysates depends on the chain length and hydrophobicity of the peptides, which depends on the hydrolysis conditions used. Leucine is one of the most abundant amino acids in enzymatic bromelain seaweed hydrolysates, which contributes to their bitter taste^80^.

### Other protein sources

In addition to the protein sources mentioned above, there are other protein sources that are also used in the food industry. These protein sources include plant-based foods (like fruits, vegetables, grains, nuts, and seeds) and animal-based foods (like meat, poultry, and eggs). Researchers have also investigated the bioactivity and bitterness of the hydrolysates produced from these proteins.

Legumes have high protein content, low levels of methionine, cysteine, and tryptophan, and high levels of lysine compared to cereals^81^. The bitterness intensity of lentil protein hydrolysates produced using chymotrypsin is unaffected by the degree of hydrolysis^82^. The selection of enzyme used to hydrolyze legume proteins impacts the bitterness of the peptides produced, with alcalase producing bitter peptides and flavourzyme producing sweet peptides from lentil proteins^83^. Other studies have also reported that alcalase produces more bitter peptides than other enzymes during the hydrolysis of pea proteins^84–87^. In contrast, Koo et al.^88^ hydrolyzed wheat gluten protein using various enzymes and reported that alcalase-derived hydrolysates exhibited the lowest bitterness, while flavourzyme- and protamex-derived ones exhibited the highest bitterness after extensive hydrolysis. In another study, Taghizadeh et al.^12^ showed that the hydrolysis of proteins from medicinal plants using pancreatin generates bitter peptides, with *Ziziphora clinopodioides* producing the bitterest ones. Mushroom protein hydrolysates derived from papain and neutrase have also been shown to have a bitter taste^89^. Kheeree et al.^90^ hydrolyzed lemon basil seed proteins using alcalase and identified two ACE inhibitory peptides, which also exhibited a bitter taste. Overall, these studies suggest that the amino acid number, type, and sequence influence the bitterness of the hydrolysates produced from different plant protein sources using different hydrolysis methods.

Animal by-products rich in high-quality proteins can be used to produce high-value functional ingredients^91^. Singh et al.^92^ showed that the hydrolysates produced from squid fin proteins using alcalase (40% hydrolysis degree) were rich in aspartic acid/asparagine and glutamic acid/glutamine, which made them bitter, especially at higher concentrations. Also, bitter peptides have been produced from the hydrolysis of the protein-rich by-products of cephalopods^93^ and shrimp^94,95^. The utilization of egg protein hydrolysates in the production of cottage cheese has been reported to increase the bitterness in the final product^96^. Hydrolysates of porcine and bovine hemoglobin have also been reported to exhibit a bitter taste, with the bitterness increasing with increasing molecular weight of the peptides produced^97,98^. Recently, Chen et al.^99^ reported bitter peptides from chicken protein hydrolysates generated using flavourzyme. In general, however, there is still a relatively poor understanding of the formation and properties of the bitter peptides generated by hydrolysis of animal by-products^100^. Synthetic bitter peptides

The bioactivity and functionality of food-derived peptides depend on their structural features, including their amino acid sequence, molecular weight, hydrophobicity, and net charge^101^. Hydrophobicity plays a vital role in this regard, as the number and location of non-polar functional groups is known to impact peptide properties^102^. The hydrophobicity of peptides can be increased to improve their bioactivity and functionality, but this can also lead to an increase in their bitterness, which is usually undesirable. Synthetic peptides have been produced to better understand the relationship between the molecular characteristics of peptides and their bioactivities, functionalities, and taste properties. For instance, Pripp and Ardö^103^ studied the relationship between angiotensin-(I)-converting enzyme (ACE) inhibition and the bitter taste of peptides and showed that peptides with high ACE inhibition activity are more bitter. Moreover, it has also been reported that trans-positioning two cationic residues in an amphiphilic fragment makes the peptides more hydrophobic and bactericidal^104^. In the case of the antioxidant activities, the presence of hydrophobic amino acids enhances the accessibility of the antioxidant peptides to hit their hydrophobic cellular targets^105^. Furthermore, the amino acid sequence and distance between the two bitter-taste-determinant sites (binding unit and stimulating unit) have been shown to affect the bitter taste of synthetic peptides^16,42^.

## Mechanism of human perception of peptides’ bitter taste

**Fig. 2:**
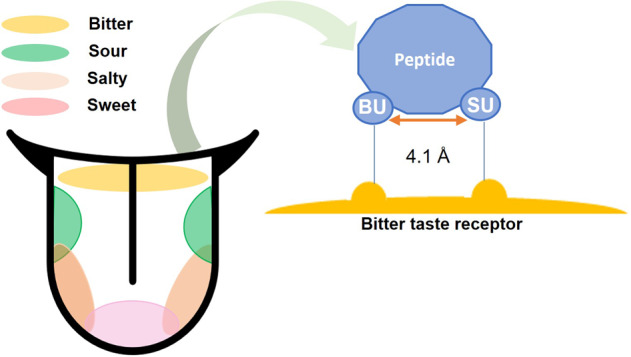
Mechanism of human perception of peptides’ bitter taste.

Peptide bitterness is perceived by humans via 25 recognized bitter taste receptors (T2Rs), which are triggered by various forms of bitter peptides^100^. As shown in Fig. 2, the generation of bitterness necessitates not just the hydrophobic characteristics, but also the crucial spatial arrangement of the peptide^33^. Peptides with a bitter taste have two functional units: a hydrophobic group with at least a backbone with three carbons called the binding unit (BU); and a basic group with α-amino group or a hydrophobic group called the stimulation unit (SU). Only when the average distance between the two sites is of approximately 4.1 Å (as in Fig. 2), the bitterness receptors detect the hydrophobicity of the bitter peptide via the hydrophobicity detection zone by starting a brain signaling cascade^106^. The bitterness intensity is related to the properties of the two determinant sites as well as the space between them. The bitter taste of peptide becomes stronger, when there are more hydrophobic groups in the hydrophobic detection zone of the bitter taste receptor. For example, Liu et al.^107^ demonstrated that the bitterness intensity of di-, tri-, and tetra-leucine was 8, 15, and 30 times greater than that of mono-leucine, respectively. This observation has been reported for tyrosine and phenylalanine as well^107^.

## Strategies to improve the palatability of bioactive peptides

To make bioactive peptides palatable there are three major strategies including debittering, taste modulating, and taste masking. It should be noted that each technique has its own benefits and drawbacks (Table 3) and may not completely remove the bitter taste.

**Table 3:** The advantages and drawbacks of various strategies for improving the palatability of bioactive peptides^27,217–221^.

Strategies		Advantages	Drawbacks
Debittering		Good debittering effects, low costs	Decrease nutritional value and biological activity, high osmolality caused by more hydrolysis
Taste masking		Does not decrease nutritional value and biological activity, prevent the dissolution of bitter substances, inhibit the binding of bitter substances with taste receptors, screening method is rapid and simple	Need a large amount of masking agent to reduce bitterness, which affects the taste and texture of the final products and increases the production costs
Encapsulation	Spray drying	Single step, continuous, high speed, high productibility, low processing costs, short processing time, good storage stability, quick and easy process, high process efficiency, microencapsulation of temperature-sensitive substances, possibility to obtain sterile products	Process at high temperature, not suitable for low water-soluble compounds, the loss of product in the walls of the drying chamber, the production of particles at the nanometer scale is limited
	Freeze-drying	Process at low temperatures and under vacuum conditions, suitable for heat-sensitive compounds, improve stability of peptides, prevent the peptide oxidation, long preservation period, minimal change in the properties, loading quantity accurate and content uniform	Time-consuming, high costs, high energy consumption
	Liposome	Entrap hydrophilic and hydrophobic peptides	The leak of trapped peptides, low physical stability, inapplicable upscaling
	Extrusion	Fast and inexpensive process	Formation of large-size capsules
	Complex coacervation	The process is simple, fast, and reproducible, the possibility of using low concentrations of surfactants, the process can be run in aqueous and anhydrous environments	Agglomeration of droplets under the influence of solvent removal, the toxicity of the solvents used
	Emulsification	Ability to encapsulate hydrophilic and hydrophobic substances, high process efficiency	Agglomeration of droplets under the influence of solvent removal, the toxicity of the solvents used
	Solid dispersion	Decrease agglomeration and release in a supersaturation state, improve wettability and increase the surface area, increase solubility and dissolution rate	Physical instability, changes in crystallinity, sensitive to temperature and humidity during storage, expensive, low reproducibility of physicochemical characteristics

### Debittering strategies

#### Treatment with active carbon, alcohol extraction, and isoelectric precipitation

**Fig. 3:**
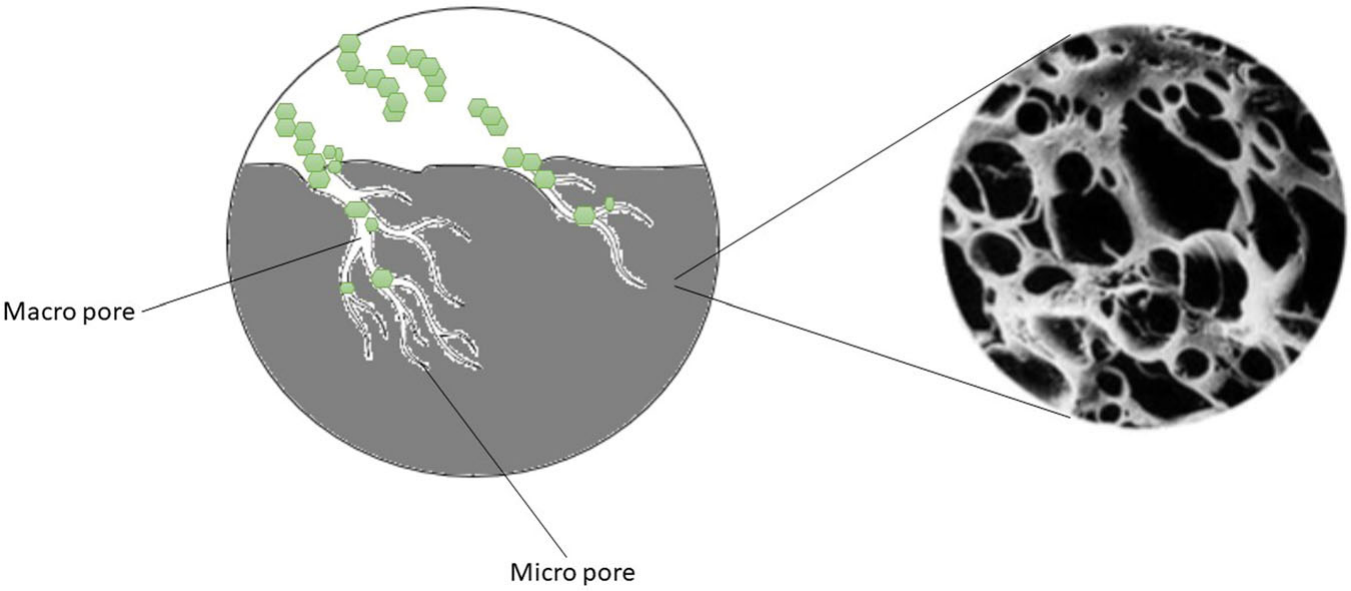
Microporous structure of activated carbon trapping bitter bioactive peptides.

Activated carbon is a good adsorbent material with a high surface area and microporous structure that can be utilized for the removal of bitter tastes (Fig. 3). However, its application does result in the loss of some desirable compounds from the hydrolysates. For instance, Cogan et al.^108^ reported that the amount of arginine (29.8%), tryptophan (63.1%), histidine (11.2%), tyrosine (24.4%), and phenylalanine (36.1%) in casein hydrolysates was significantly reduced after treatment with activated carbon. It has also been reported that activated carbon can decrease the bitterness of corn gluten hydrolysates, but their ACE inhibitory activity was also reduced^109^. In general, activated carbon is a relatively non-selective method that absorbs both non-bitter and bitter peptides. Therefore, active carbon treatments have been reported to decrease the total protein content, as well as the essential amino acid and bioactive peptide contents, thereby leading to a decrease in biological activity.

While activated carbon is generally recognized as safe^110^, there are some potential safety concerns that should be noted. One concern is that activated carbon can adsorb not only impurities, but also beneficial nutrients^108^. In addition, some types of activated carbon can contain trace amounts of impurities, such as polycyclic aromatic hydrocarbons, which could potentially pose a risk to human health^111^. Therefore, it is important to use activated carbon that is specifically intended for food use and to ensure that it meets appropriate safety standards. Overall, when used appropriately and with all safety measures in place, activated carbon can be considered as a safe and effective way to achieve the debittering of protein hydrolysates.

Some alcohols can be used as solvents to selectively extract bitter peptides from complex mixtures. The most common alcohols used for this purpose include 2-butanol, isopropyl, and ethanol^112^. Bitter peptides from *Setaria italica* protein hydrolysates have been extracted using ethanol, which improved the functional performance of the final product^113^. Liu et al.^114^ proposed that the amino acid composition and molecular weight distribution were more important factors influencing the bitterness intensity than the amino acid sequence. These authors produced wheat gluten protein hydrolysates using proteax and then debittered them using isopropyl alcohol. Their results showed that different isopropyl-extracted peptide fractions (180–500, 500–1000, and 1000–3000 Da) had different bitter tastes, with the bitterness increasing with molecular weight. In another study, it was shown that a 2-butanol solvent extraction reduced the bitterness of fish protein hydrolysates^115^. Recently, Sinthusamran et al.^116^ hydrolyzed salmon frame proteins using alcalase and flavourzyme and then debittered them using 2-butanol and isopropanol. Of the two solvents used, the hydrolysates treated with 2-butanol had the lowest bitterness. But these hydrolysates also had a reduced antioxidant activity. Salmon frame hydrolysates have also been obtained using alcalase and then debittered using 2-butanol followed by β-cyclodextrin treatment^117,118^. This combined treatment led to hydrolysates that exhibited no bitter taste. The debittered hydrolysates were then used to fortify biscuits without introducing a strong bitter taste.

Isoelectric precipitation can also be applied to reduce the bitterness of protein hydrolysates. In this case, the bitter peptides are selectively precipitated by adjusting the pH to around their isoelectric points. Adler‐Nissen^119^ showed that the bitterness of soy protein hydrolysates was reduced after precipitation of bitter hydrophobic peptides around their isoelectric pH.

In summary, these debittering methods are usually relatively simple and inexpensive to apply. However, they may remove some desirable peptides from the hydrolysates, and sometimes involve the utilization of organic solvents, which is often undesirable from a cost and sustainability perspective.

#### Chromatographic separation

Depending on the nature of the resins used, hydrophobic bitter peptides can be separated from complex mixtures using chromatographic methods. The most commonly used resins for debittering are dextran gel, hexyl-gel, and microporous adsorption-resins^114^. Kim et al.^120^ isolated bitter peptides from tryptic hydrolysates of 11S glycinin using RP-C18 HPLC, with the final hydrolysates showing a hydrophobicity below 1400 cal/mol. In another study, Liu et al.^121^ isolated four bitter peptides from whey protein hydrolysates using ultrafiltration (3 kDa cut off) and RP-C18 column chromatography. Initially, the authors showed that the peptide fraction less than 3 kDa had a bitter taste, while the peptide fraction more than 3 kDa was tasteless. Afterward, the lower molecular weight fraction was removed using a C18 column, and 4 peptides were identified as having the strongest bitter taste. A Sephadex G-25 column has been successfully used to remove bitter peptides from skim milk protein hydrolysates by Helbig et al.^122^. Silica gel chromatography using propyl alcohol as a solvent has also been used to eliminate bitter peptides from casein and cheese hydrolysates^123^. Ultrafiltration can also be used to remove bitter peptides based on differences in the molecular weights of bitter and non-bitter peptides. For instance, Aubes-Dafau et al.^97^ debittered hemoglobin hydrolysates using ultrafiltration. These authors showed that peptides ranging from 500 to 5000 Da were very bitter. Dauksas et al.^115^ used cholestyramine resin, a strong anion exchange resin, to isolate bitter peptides from fish protein hydrolysates. The authors indicated that the bitter intensity was reduced more than with the use of 2-butanol treatment. Cheison et al.^124^ used a macroporous adsorption resin for simultaneous desalting and debittering of whey protein hydrolysates. The authors hydrolyzed whey protein isolate using protease N ‘Amano’ G (IUB 3.4.24.28) and after loading the hydrolysates on the resin, the compounds were eluted using 20%, 40%, and 75% alcohol solutions. The results showed that the fraction eluted using 75% alcohol was highly bitter, exhibited strong ACE inhibition, had the highest content of hydrophobic amino acids, and was comprised of approximately 71% peptides below 600 Da. Imai et al.^125^ created a new type of silica resin without chemical modifications for adsorption of non-polar and cationic peptides through hydrophobic and electrical interactions. Imai et al.^126^ then showed that this resin was effective at removing bitter peptides.

#### Additional hydrolysis

Considerable efforts have been focused on using peptidases and proteases for reducing the bitter taste of protein hydrolysates. It has been reported that the simultaneous or sequential incubation of hydrolysates with various enzymes can selectively release hydrophobic residues from bitter peptides. The most commonly used enzymes for debittering purposes are exopeptidases, endopeptidases, and proteases^19^. Since the bitter taste of peptides arises from the existence of branched or hydrophobic residues that may be located at the N- or C-terminal positions, further hydrolysis can be performed using an aminopeptidase or carboxypeptidase, respectively^127^. However, it is important that this further hydrolysis does not adversely affect the bioactivity and functional properties of the peptides formed^128^. Moreover, the additional costs and time involved may limit the large-scale utilization of this approach for the industrial production of debittered peptides^129^.

The effectiveness of peptide debittering depends on the type of enzyme used. Several aminopeptidases from *Thermophilic* and *Aspergillus* fungi have been shown to have a strong specificity for cleaving hydrophobic and aromatic amino acids at the N‐terminal of peptides^130–132^. Researchers have used enzymes such as thermostable leucine aminopeptidase from *Thermomyces lanuginosus* and *Aspergillus niger*^131^, and aminopeptidase AN-APA from *A. niger* CICIM F0215 to debitter soy, casein, milk, and cheese hydrolysates by hydrolyzing hydrophobic and hydrophilic residues at the N-terminal of polypeptides^132^. Aminopeptidases from *Aeromonas caviae* T-64, *Lactococcus lactis* subsp. cremoris WG2 or AM2, *Lactococcus lactis* subsp. Cremoris, *Lactobacilli helveticus* strain L1, *Penicillum caseicolum*, *Pseudomonas fluorescens* ATCC 948, *A. niger*, and cotyledon of soybean have also been successfully used to debitter milk and cheese hydrolysates^19,133–141^. Nishiwaki et al.^142^ indicated that the treatment of bitter soy hydrolysates with an aminopeptidase from *Grifola frondosa* led to the release of hydrophobic amino acids, such as valine, leucine, phenylalanine, tyrosine, and isoleucine, which decreased the bitterness of the hydrolysates. In addition, Li et al.^143^ hydrolyzed soybean proteins using alcalase followed by further hydrolysis using an exopeptidase from *Actinomucor elegans*, to reduce their bitterness. Corolase is a commercial enzyme mixture that contains aminopeptidase, elastase, chymotrypsin, tryptic, and dipeptidase activities. It has been used for hydrolysis of both animal and vegetable proteins as it provides a high degree of hydrolysis without producing bitter peptides^127^. Accelase is an aminopeptidase from *Lactococcus lactis* that can be applied to prevent bitterness during cheese ripening. Debitrase is another aminopeptidase from *Lactococcus lactis* and *Aspegillus oryzae* that can be used to reduce the bitterness caused by conventional enzymatic hydrolysis^127,144^. Protease M is an enzyme mix from *A. oryzae* that has a pepsin-like endopeptidase activity coupled with carboxyl, amino, and leucine exopeptidase activities. Hinnenkamp and Ismail^145^ recently showed that protease M releases a greater proportion of hydrophilic peptides upon hydrolysis of whey proteins, resulting in a decrease in bitterness. Furthermore, alcalase-treated anchovy hydrolysate was treated with an aminopeptidase derived from the common squid *Todarodes pacificus* hepatopancreas to reduce bitterness^146^. Yan et al.^147^ expressed *Streptomyces canus* T20 aminopeptidase (ScAPase) in *Bacillus subtilis* followed by an additional treatment of trypsin-hydrolyzed rice protein isolates previously treated with recombinant ScAPase. Their results showed that hydrolysates could be produced that did not exhibit bitterness but did exhibit high ACE inhibitory and antioxidant activities. Tong et al.^24^ hydrolyzed soybean proteins using protex 6 L followed by protease A 2 SD, which is an enzyme that cleaves leucine and arginine at the N-terminal of bitter peptides. This combined treatment led to the production of low bitter hydrolysates having high antioxidant activity.

Hydrolysis of corn gluten using a pescalase and protease A mixture was shown to reduce the bitterness of hydrolysates more than a flavourzyme treatment^148^. In another study, a leucine aminopeptidase Lap1 from *Aspergillus sojae* GIM3.30 was overexpressed in *Pichia pastoris*^149^. This enzyme was then used to produce casein and soy protein hydrolysates with low bitterness. Fu et al.^150^ hydrolyzed minced beef and porcine plasma using 10 food-grade enzymes, including protease A, protease P, proteAX, flavourzyme, alcalase, papain, bromelain, protamex, neutrase, and sumizyme BNP-L. Among these, the protease A generated hydrolysates with the lowest bitterness.

Several studies have demonstrated the ability of carboxypeptidases to debitter protein hydrolysates. Carboxypeptidases from *Actinomucor elegans* were able to reduce the bitterness of soybean protein hydrolysates during incubation at 40–95 °C for 6 h^151^. Kawabata et al.^152^ further hydrolyzed soybean and gluten hydrolysates derived from pepsin and trypsin using carboxypeptidase from squid liver, leading to decreased bitterness levels. The same goal has been successfully performed on peptic hydrolysates of soybean proteins, peptic hydrolysates of fish protein concentrate, and casein hydrolysates using wheat carboxylases^153–155^. There are many carboxypeptidases available for debittering protein hydrolysates^127^. Recently, it was reported that *Bacillus subtilis* ACCC 01746 was able to produce proteases and carboxypeptidases in the early stage of solid-state fermentation of soybean meal^156^. These enzymes were shown to hydrolyze soybean proteins and produce peptides with only a mild bitterness.

Meinlschmidt et al.^157^ fermented soy protein hydrolysates using *Lactobacillus perolens*, *Rhizopus oryzae*, and *Actinomucor elegans* and showed that all strains decreased the bitterness to a minimum level (0.7) in comparison with non-fermented hydrolysates (2.8–8.0) and untreated soy protein isolate (2.8). The bitterness scale used ranged from no perception (0) to strong perception (10).

In addition to peptidases, alkaline/neutral proteases have also been used for debittering protein hydrolysates. Kodera et al.^158^ hydrolyzed soy proteins using various enzymes, especially protease D3. This enzyme was purified from germinated soy cotyledons and used to generate hydrolysates with low bitterness levels. In another study, tryptic β-casein hydrolysates were hydrolyzed using Xaa-Pro dipeptidyl peptidase, a prolyl aminopeptidase from *Lactobacillus casei ssp. casei* LLG, resulting in a decrease in the bitterness of proline-rich peptides and other peptides containing proline^159^. Moreover, the hydrolysis of the by-product of silver carp was also performed using different proteases^160^. The results indicated that hydrolysis using a combination of neutrase and flavourzyme generated hydrolysates with lower bitterness compared to the other proteases studied. Recently, Zhang et al.^161^ demonstrated that the proteases isolated from soybean seedlings can completely remove the bitterness of soy protein hydrolysates derived from alcalase 2.4 L, without reducing the antioxidant activity of the final products. In addition, the hydrolysis of soybean proteins using a mixture of tripeptidase and alkaline protease not only decreased the bitterness but also improved the functional properties of the hydrolysates^162^. Hydrolysis of wheat gluten using proteax for 300 min has been reported to generate hydrolysates ranging from 180 to 500 Da, which had a low bitterness level^107^. A combination of alkaline protease hydrolysis and transglutaminase cross-linking was able to rearrange the polypeptide structure of soybean protein hydrolysates, resulting in products with reduced bitterness^48^.

According to these findings, additional hydrolytic processes can be effectively used to reduce the bitterness of protein hydrolysates and to transform them into valuable food ingredients. However, the extent of this additional hydrolysis has to be optimized to produce peptides that maintain their desirable functional attributes. For example, Cheung et al.^128^ revealed that further hydrolysis of whey protein hydrolysates with exopeptidases could significantly decrease the bitterness, while the ACE inhibitory activity of the resultant hydrolysates dramatically decreased from an IC_50_ of 0.15 mg/mL to 0.34 mg/mL as well. The authors illustrated that this was due to the fragmentation of active peptides, which resulted in lower ACE-inhibitory activity.

### Bitterness inhibitors: masking and blocking strategies

Bitterness inhibitors can mask the bitter taste of peptides. These inhibitors include additives that can mask, block, or modify the taste of bitter peptides. However, the use of these inhibitors is sometimes limited due to their high cost and off flavors^129^.

The most commonly used masking agents include additives like modified starch, taurine, glycine, polyphosphates, as well as additives that chemically modify peptides through amination, deamination, acetylation, or cross-linking procedures^129^. A good example of this phenomenon is the Maillard reaction, which involves interactions between the carbonyl groups of reducing sugars and the free amine group of peptides^100^. Eric et al.^163^ reported that the taste and antioxidant activity of sunflower protein hydrolysates improved by using the Maillard reaction by heating the peptides in the presence of xylose and cysteine at 120 °C for 2 h. In addition, using transglutaminase to crosslink the Maillard reaction products generated from soybean protein hydrolysates (120 °C for 2 h) has been shown to reduce bitterness and generate savory tastes^164^. In contrast, de Carvalho et al.^165^ showed that transglutaminase cross-linking of whey protein hydrolysates did not alter their bitterness. This effect may be explained by the presence of free glutamine and the considerable number of short peptides without glutamine or lysine residues. However, glycation of poultry protein hydrolysates using glucosamine in the presence of microbial transglutaminase resulted in an enhanced salty taste of the hydrolysates^166^. Deamination of wheat gluten hydrolysates (37 °C for 1.5–3 h) was reported to decrease the bitterness intensity of the hydrolysates^167^. Moreover, acetylation of lysine in bitter peptides can decrease their bitterness. Won Yeom et al.^168^ acetylated lysine and tyrosine of soybean hydrolysates using N-acetyl imidazole and then deacetylated the *O*-acetyl tyrosine at pH 11, resulting in a decrease in the bitterness of the hydrolysates. Recently, Habinshuti et al.^169^ characterized the taste and antioxidant activity of Maillard reaction products from hydrolysates of sweet potato, potato, soy isolate, egg white, and whey isolate proteins. The authors reported that the Maillard reaction increased the sweetness, sourness, and umami tastes and decreased the bitterness of all the hydrolysates. However, the Maillard reaction products from the whey hydrolysates exhibited the strongest oxygen radical absorbance capacity. Abdelhedi et al.^170^ hydrolyzed smooth hound viscera protein using neutrase, esperase, and purafect followed by a Maillard reaction of the low molecular peptides produced (<1 kDa) in the presence of sucrose (90 °C for 2 h). The results showed that the glycation degree was considerably enhanced in the esperase-derived peptides/sucrose conjugates, which led to a reduction of the bitter taste and enhancement of the antioxidant activities of the hydrolysates.

The most common blocking agents currently used include sodium salts, phospholipids, neohesperidin, zinc lactate, ferulic acid, γ-aminobutyric acid, β-lactoglobulin, monosodium glutamate, and adenosine monophosphate^129,171^. Xu et al.^172^ demonstrated that sodium chloride suppressed the bitter taste of egg white and chicken protein hydrolysates in a concentration-dependent manner. The authors stated that adding sodium chloride at certain concentrations led to a salting-in effect, which buried the hydrophobic groups, decreased the surface hydrophobicity of the peptides, and resulted in a decreased bitterness. Therefore, this strategy is an alternative, effective, and cheap method to suppress the bitterness of protein hydrolysates. Dong et al.^173^ prepared a complex of neohesperidin dihydrochalcone and glucosyl-β-cyclodextrin to block the bitterness of corn peptides. The complex formed had a better bitterness-masking effect than single neohesperidin dihydrochalcone or glucosyl-β-cyclodextrin, phosphatidic acid, protein-phosphatidic acid complexes, tannin, and neodiosmin. Zhang et al.^174^ hydrolyzed beef protein using 6 commercial enzymes including alcalase, chymotrypsin, trypsin, pepsin, flavourzyme, and thermoase, and determined the ability of these enzymes to block the T2R4 bitter taste receptor. The results showed that the treatment of the HEK293T cells expressing the T2R4 receptor with hydrolysates effectively reduced calcium mobilization, resulting in a reduction of the bitterness intensity. In addition, Xu et al.^175^ demonstrated that chicken protein-derived peptides are able to block human bitter taste receptors of T2R4, T2R7, and T2R14.

Other agents, such as salts, sweeteners, and aromatic agents can also be used as taste-correcting agents to reduce bitterness^176,177^. For instance, Leksrisompong et al.^178^ added 24 bitter taste inhibitors to whey protein hydrolysates and found that sucralose, fructose, sucrose, adenosine 5’-monophosphate (5’AMP), adenosine 5’-monophosphate disodium (5’AMP Na_2_), sodium acetate, monosodium glutamate, and sodium gluconate were effective bitter taste inhibitors. The authors also reported that sodium chloride was an effective inhibitor of the bitter taste of hydrolysates containing small peptides, but it was not effective for those containing large peptides. Furthermore, they showed that lysine and arginine could inhibit the bitter taste of quinine, but not the whey hydrolysates.

### Encapsulation technologies

**Fig. 4:**
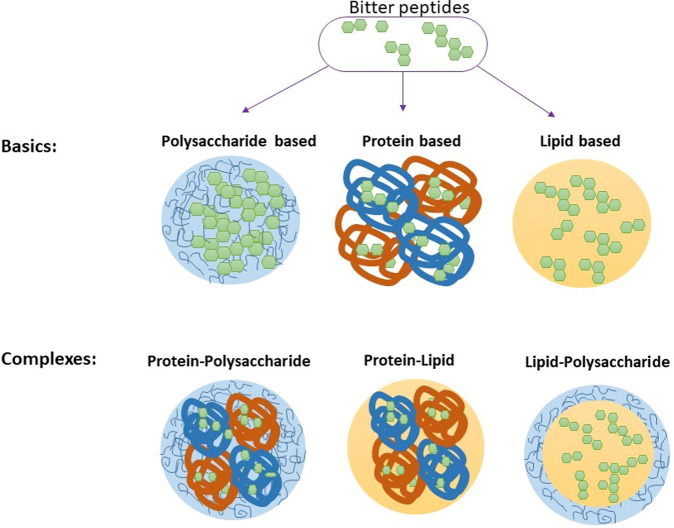
Schematic representation of various polymer-based encapsulation systems.

Encapsulation is widely used to improve the dispersibility, stability, and bioactivity of protein hydrolysates, but it can also be used to reduce the bitterness of protein hydrolysates and peptides (Table 4). Encapsulation technologies are often characterized as either micro- or nano-encapsulation depending on the dimensions of the particles used. Nano-encapsulation utilizes particles with dimensions below about 1000 nm, whereas Micro-encapsulation utilizes particles with dimensions above this value. For oral administration applications, nano-encapsulation is often more suitable because it has less impact on the mouthfeel of ingested substances, and leads to products that are more resistant to gravitational separation and aggregation^179^. The particles used to encapsulate bioactive peptides can be assembled entirely from food-grade ingredients, such as polysaccharides, proteins, and lipids (Fig. 4) using a range of techniques including spray drying, freeze drying, complex coacervation, emulsification, antisolvent precipitation, extrusion, electro-spinning, electro-spraying, liposome, and solid dispersion technologies^179^. Factors that can affect the encapsulation efficiency include the peptide characteristics (molar mass, polarity, and charge), particle characteristics (composition, size, shape, internal/external polarity, and interfacial properties), and production conditions (method and operating conditions)^180^.

**Table 4:** Summary of the main carriers and technologies used for the encapsulation of protein hydrolysates and peptides for masking their bitter taste.

Technologies	Carriers	Hydrolysates/peptides	References
Spray drying	Maltodextrin	Casein hydrolysates	^222^
Spray drying	Maltodextrin/β-cyclodextrin	Whey protein hydrolysates	^223^
Spray drying	Maltodextrin and gum arabic	Chicken meat protein hydrolysate	^224^
Spray drying	Gum arabic	Casein hydrolysates	^225^
Spray drying	Maltodextrin or maltodextrin/β-cyclodextrin	Whey protein hydrolysates	^223^
Spray drying	Gelatin/soy protein isolate	Casein hydrolysates	^44^
Spray drying	Chitosan and gelatin	Whey protein hydrolysates	^226^
Spray drying	γ-cyclodextrin	Whey protein hydrolysates	^227^
Spray drying	Soy protein isolate	Casein hydrolysates	^228^
Spray drying	Maltodextrin	Flaxseed protein hydrolysates	^229^
Spray drying/freeze drying	Whey protein concentrate/sodium alginate	Whey protein hydrolysates	^181^
Spray drying/freeze drying	Maltodextrin	Soybean hydrolysates	^230^
Spray drying/freeze drying	Chitosan/lecithin	Oyster protein hydrolysates	^231^
Freeze drying	Maltodextrin/gum arabic	Casein hydrolysates	^231^
Complex coacervation	Soy protein hydrolysates/pectin	Casein hydrolysates	^232^
Complex coacervation	Whey protein/gum Arabic	Bean protein hydrolysates	^233^
Complex coacervation	Pectin	Whey protein hydrolysates	^234^
Solid dispersion	Chitosan	Rainbow trout skin gelatin hydrolysates	^235^
Solid dispersion	Chitosan	Salmon protein hydrolysates	^187^
Solid dispersion/spray-drying	Gum arabic and maltodextrin	Pea protein isolate	^236^
Emulsification	Polyglycerol polyricinoleate	Soy hydrolysates	^237^
Emulsification	Polyglycerol polyricinoleate/alginate	Oyster peptides	^238^
Emulsification	Whey protein concentrate/inulin/fucoidan	Fish protein hydrolysate	^239^
Extrusion	Alginate	Whey protein hydrolysates	^240^
Extrusion	Alginate/collagen, alginate/arabic gum, and alginate/gelatin	Whey protein hydrolysate	^241^
Extrusion and emulsification	Sodium alginate-*O*-carboxymethyl chitosan	ACE inhibitory peptides	^242^

Spray-drying is currently the most widely used method to encapsulate bitter peptides due to its ease of use, low cost, and scalability^179,181^. This method typically involves spraying a fluid polymer solution or colloidal dispersion containing the peptides through a nozzle into a high temperature chamber. The water is rapidly evaporated leading to the formation of solid microparticles that contain the peptides trapped within a polymer and/or colloidal matrix, which is usually referred to as the wall material. Proteins and carbohydrates (oligosaccharides or polysaccharides) are often utilized as natural polymers that act as wall materials. For instance, Sarabandi et al.^182^ used spray drying to encapsulate casein hydrolysates using maltodextrin (DE = 18) as a wall material. Their results showed that the bitterness of the hydrolysates was reduced, while the antioxidant activity was preserved. Murthy et al.^183^ prepared a vegetable soup enriched with microencapsulated pink perch meat protein hydrolysates using spray drying and maltodextrin/arabic gum as a wall material. Sensory analysis showed that the bitterness of this product was less than that of a soup containing non-encapsulated hydrolysates. Spray-drying has also been used to encapsulate casein and whey hydrolysates using various other kinds of wall material including soy protein isolate, maltodextrin, maltodextrin-β-cyclodextrin, sodium alginate, whey protein concentrate, γ-cyclodextrin, arabic gum, and gelatin^184,185^.

In some cases, emulsion technology can be used to encapsulate peptides^179^. For instance, hydrophobic peptides can be dispersed within an oil phase, which is then homogenized to form an oil-in-water (O/W) emulsion or nanoemulsion containing peptide-loaded oil droplets. Alternatively, more hydrophilic peptides can be trapped within the water droplets in water-in-oil (W/O) emulsions. As an example, the encapsulation of bitter peptides in a high internal phase W/O emulsion was reported to greatly reduce the bitterness but increase the gastrointestinal stability of the peptides^186^. Liposome technology can also be used to encapsulate peptides and reduce their bitterness. The efficacy of liposome encapsulation has been shown to depend on the molecular weight, polarity, and charge of the peptides. For instance, Li et al.^187^ encapsulated Atlantic salmon protein hydrolysates in chitosan-coated liposomes at a high encapsulation efficiency (71%). Similarly, marine protein hydrolysates (*Micropogonias furnieri* muscle and by-product) have been incorporated into liposomes at a high encapsulation efficiency (80%)^188,189^ and whey peptides have been incorporated into soy lecithin liposomes at a high encapsulation efficiency (90%)^190^. Conversely, other researchers have reported a relatively low encapsulation efficiency (40–60%) for casein peptides in liposomes, which was mainly attributed to an electrostatic repulsion between the anionic casein phosphopeptides and the anionic head groups of the phospholipids used to assemble the liposomes^180^. Another study reported that even though the encapsulation efficiency (48%) and antioxidant activity of whey peptides in phosphatidylcholine liposomes were relatively low, the antioxidant activity of the encapsulated peptides was higher than that of the non-encapsulated ones after 30 days storage^191^. The encapsulation of other dairy protein hydrolysates has recently been reviewed by Giroldi et al.^185^.

Biopolymer-based microgels have also been shown to be suitable for encapsulating bitter peptides. Han et al.^192^ designed a polysaccharide-based encapsulation system to encapsulate peanut peptides through electrostatic complexation of cationic n-trimethy chitosan and anionic alginate. The authors reported that the antioxidant activity of the peptides increased after encapsulation. They also reported that the peptides were retained within the complexes under simulated gastric conditions but released under simulated small intestinal conditions. Moreover, the authors reported that this complex was biocompatible and nontoxic, which makes it suitable as a delivery system for peptides.

The bitterness of some peptides can also be reduced by forming inclusion complexes with cyclodextrins, which have a hydrophobic cavity and a hydrophilic exterior^193^. Hydrophobic peptides can be incorporated into the cavity through a guest-host interaction, which reduces their tendency to interact with the bitter receptors in the mouth. For instance, Xia et al.^87^ compared various methods of reducing the bitterness of pea protein hydrolysates, including flavourzyme treatment, butanol extraction, and encapsulation using β-cyclodextrin. The butanol extraction and β-cyclodextrin encapsulation methods led to the lowest bitterness score. Numerous other studies have shown that the bitterness of hydrophobic and aromatic amino acids can be greatly reduced by forming inclusion complexes with cyclodextrin^194,195^. As an example, Hou et al.^196^ reported that β-cyclodextrin interacted with hydrophobic peptides, thereby reducing the bitterness of soybean protein hydrolysates. Sim et al.^197^ also reported that the bitterness of soybean peptides could be reduced by forming inclusion complexes with β-cyclodextrin.

## Conclusions and future perspectives

Many protein hydrolysates and bioactive peptides have been described as functional compounds with one or more biological activities that could potentially improve the health of the population. However, it is often challenging to create functional foods fortified with these bioactive substances because of their bitterness. The perceived bitterness of bioactive peptides depends on the number, type, and sequence of amino acids they contain, which is governed by the protein source, hydrolysis method, and operating conditions. The nature of the side chains on the peptides formed by hydrolysis is mostly responsible for peptide bitterness. The distribution and placement of bitter taste residues, hydrophobicity, the degree of hydrolysis, amino acid conformation, peptide sequence, and the amount of carbon atoms on the side chains of amino acids are other factors that influence the bitterness of protein hydrolysates. Practical approaches are therefore required to reduce the bitterness of protein hydrolysates and peptides, without adversely affecting their beneficial biological activities. Techniques such as the use of active carbon have proven to be beneficial, although they resulted in the loss of some desirable compounds from the hydrolysates and their associated biological activities. Thus, it is necessary to research further and design functional materials that can specifically bind bitter peptides without having a negative impact on the concentration of other peptides or active molecules in order to preserve the biological activities if these compounds.

A variety of strategies have been developed to reduce the bitterness of bioactive peptides, which vary in their efficacy and potential for commercial applications. Debittering and taste masking are two approaches that have been considered as the primary solutions of bitter taste of peptides. However, encapsulation technologies are one of the most promising strategies that have been developed for this purpose. Bitter peptides can be trapped in various kinds of colloidal particles, which can improve their water dispersibility, stability, and bioactivity, as well as reducing their bitterness by preventing them from interacting with bitter receptors in the mouth. At present, however, there is still a relatively poor understanding of the efficacy of different kinds of encapsulation technologies for specific applications, which makes it difficult to select the most appropriate one. Finally, further research is needed in this field in order to create methods that are ideally accessible, scalable, and effective for the oral delivery of biologically active peptides, as well as a standardization of the testing conditions using each technology, to clearly elucidate the efficiency of each method and their industrial scale-up, allowing ultimately the development of palatable and more nutritious food products.

### Reporting summary

Further information on research design is available in the Nature Research Reporting Summary linked to this article.

## Supplementary information

Reporting Summary

## Data Availability

All the data used in this research is described in the manuscript.
